# Clinical Significance of Renal Tissue in Neonatal Sacrococcygeal Teratoma: A Case Report With Review of Literature

**DOI:** 10.1177/10935266261440599

**Published:** 2026-04-24

**Authors:** Alessio Conte, Silvia Rebella, Gabriele Gaggero, Valerio Gaetano Vellone, Massimo Conte, Francesca Parisi, Federico Palo, Marco Pavanello, Alberto Giardina, Elena Arkangelskaya, Stefania Sorrentino

**Affiliations:** 1 Department of Neuroscience, Rehabilitation, Ophthalmology, Genetics, Maternal and Child Health, University of Genoa, Italy; 2 Pathology Unit, IRCCS Istituto Giannina Gaslini, Genoa, Italy; 3 Department of Surgical Sciences and Integrated Diagnostic (DISC), University of Genoa, Italy; 4 Oncology Unit, IRCCS Giannina Gaslini, Genoa, Italy; 5 Pediatric Surgery Department, IRCCS Istituto Giannina Gaslini, Genoa, Italy; 6 Division of Neurosurgery, IRCCS Istituto Giannina Gaslini, Genoa, Italy; 7 Neonatal and Pediatric Intensive Care Unit, Acceptance and Emergency Department, IRCCS Istituto Giannina Gaslini, Genoa, Italy; 8 Pediatric Radiology Department, IRCCS Istituto Giannina Gaslini, Genoa, Italy

**Keywords:** sacrococcygeal teratoma, congenital teratoma, Wilms tumor, ectopic renal tissue

## Abstract

Congenital sacrococcygeal teratomas (SCTs) are rare tumors with highly variable prognosis, influenced by associated abnormalities and marked histological heterogeneity. SCTs may exhibit somatic renal differentiation, encompassing a wide spectrum of cytological and architectural features, for which accepted diagnostic criteria are lacking. This renders the distinction between immature nephrogenic tissue and true nephroblastoma particularly challenging. We report an illustrative neonatal case of SCT containing a minor nephroblastomatous component and conducted a systematic review to evaluate management and outcomes in newborns (≤28 days old) with renal tissue identified within SCTs. A comprehensive search of PubMed, Scopus, and Web of Science yielded 532 records, of which 16 studies met inclusion criteria. Including the present case, 19 newborns were analyzed. Renal tissue was described as immature or ectopic nephrogenic tissue in 15 cases (79%) and as overtly malignant, consistent with Wilms tumor, in 4 cases (21%). All patients underwent surgical resection, while chemotherapy (n = 5; 26.3%) and radiotherapy (n = 2; 10.5%) were less frequently administered. Median follow-up was 29.5 months (range: 4-154), with no cancer-related mortality. In the absence of standardized diagnostic criteria, renal differentiation within SCTs represents a diagnostic and therapeutic dilemma, supporting a cautious, multidisciplinary management approach.

## Background

Teratomas account for approximately 29% of congenital neoplasms, with 45% of cases occurring at the sacrococcygeal region.^1^ The incidence of sacrococcygeal teratomas (SCTs) is estimated at 1in 35 000 live births, with a female-to-male ratio of 4:1. Approximately 80% of SCTs are benign,^2^ particularly when arising congenitally. Following the migration of the germ cell ridge, teratomas can develop virtually anywhere along the body midline. SCTs arise in the presacral space, between the upper two-thirds of the rectum and the sacrum.^3^ SCTs may be associated with urogenital abnormalities, central nervous system lesions and genetic syndromes as Klinefelter, trisomy 13 and 21, Beckwith-Wiedemann, and Proteus syndrome.^4^

The American Academy of Pediatrics has classified SCTs into 4 subtypes based on their location relative to the pelvis: predominantly external, predominantly external with a significant intrapelvic component, predominantly internal and entirely internal.^5^ The overall mortality rate for SCTs is approximately 5% when diagnosed at birth but increases up to 50% when diagnosed prenatally.^6^ In the context of SCTs, it is possible to identify malignant degeneration of the somatic components. In particular, the degeneration of renal somatic components leading to the development of Wilms tumor has been described.^7-10^ Wilms tumor (WT), or nephroblastoma, is the most common pediatric renal tumor.^3^ WT arise due to incomplete mesenchymal-epithelial transition (MET) with aberrant proliferation of pluripotent cells leading to tumorigenesis.^3^ Histologically, WT is composed of epithelial, mesenchymal, and stromal elements similar to those of normal renal structures.

Extrarenal Wilms tumor (ERWT) is a rare entity accounting for approximately 0.5% to 1% of all WT cases.^11^

Similarly, the presence of ectopic immature renal tissue (EIRT) has rarely been described,^12^ including within teratomas. Several terms have been used to describe this entity, such as “ectopic nephrogenic rests”^13^ (ENR) or “extrarenal nephroblastomatosis.” It is proposed that these lesions result from aberrant localization of metanephric remnants during fetal development.^12^

Immature somatic nephrogenic tissue can rarely be found within SCTs, posing unique challenges in histological diagnosis and clinical management. Given the rarity of these cases, there are currently no universally accepted treatment guidelines. Consequently, management decisions are based on individualized risk assessment and may range from surgical resection alone to the addition of chemotherapy and/or radiotherapy.

## Case Report

A 38-week-old female neonate was delivered via cesarean section. The newborn exhibited a large subcutaneous sacral nodule of soft consistency with intact overlying skin and anterior displacement of the anus. Family history was unremarkable. The lesion was initially identified via prenatal ultrasound at 29th week of gestation. Subsequently, prenatal magnetic resonance imaging (MRI) revealed a sacrococcygeal lesion compatible with teratoma (measuring 8 cm × 5 cm × 6.5 cm), demonstrating both extra- and intraspinal extension with subtotal spinal canal involvement up to the D8 vertebral level and no clear margins (Figure 1).

**Figure 1.**
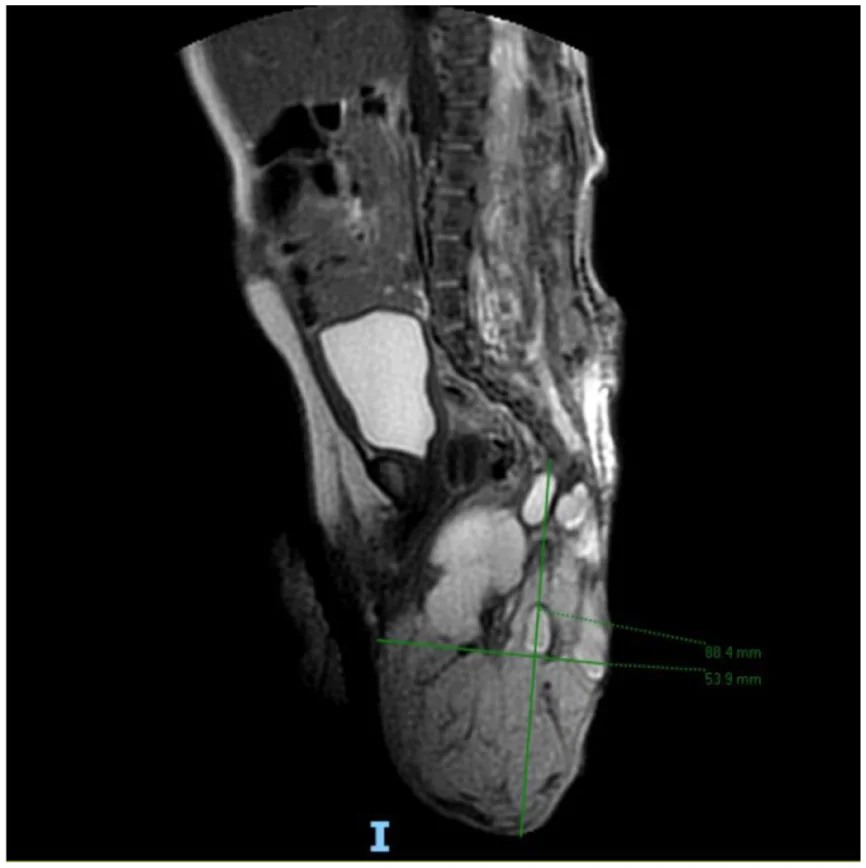
MRI at diagnosis, showing sacrococcygeal lesion with intra- and extraspinal extension.

Neurological examination revealed a reduction in right lower limb reflexes; no clear sphincter dysfunction was noted. Abdominal ultrasound did not reveal additional lesions or malformations. Serum alpha-fetoprotein (AFP) concentration was 55 200 IU/ml, while beta-hCG (β-hCG) was 12 mU/ml. Staging investigations excluded metastatic disease. An emergency laminotomy was performed on the fifth day of life to decompress the spinal cord. The intraspinal tumor was only partially excised due to massive involvement of the nerve roots. Macroscopically, the specimen exhibited a solid lobulated structure with translucent and hemorrhagic areas. A peripheral minor solid nodule was evident (Figure 2(A)). Microscopic examination revealed an admixture of mesenchymal, ectodermal, and endodermal components, predominantly mature (approximately 95%). Minor component (approximately 5%) consisted of immature, roundish elements with poor cytoplasm and a compact, blastematous-appearing architecture, intermixed with poorly differentiated tubular structures and rudimentary renal glomeruli (Figure 2(B)-(E)).

**Figure 2.**
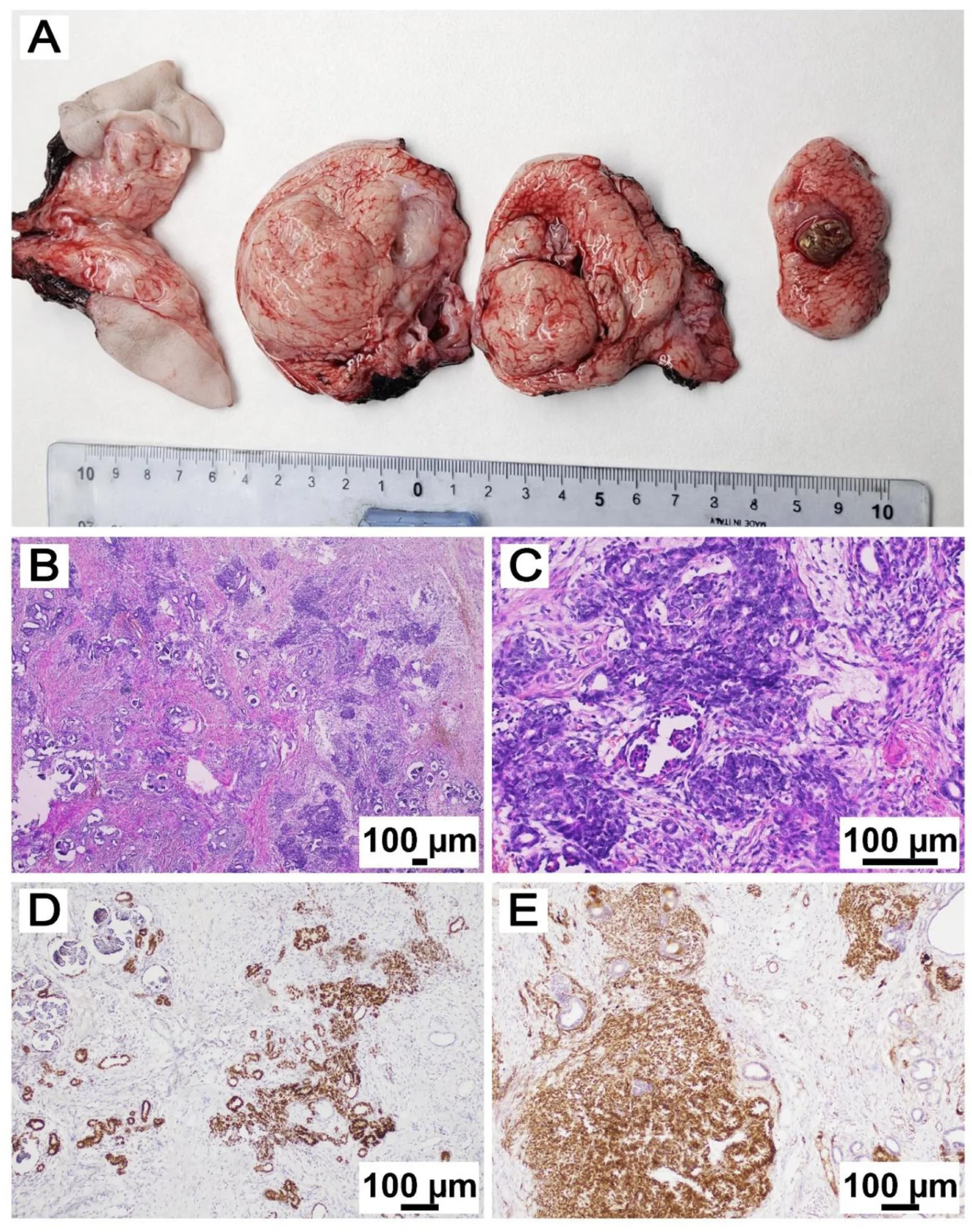
Histological evaluation of tumor specimen. (A) Gross appearance of the sacrococcygeal teratoma, showing multiple lobulated fragments with heterogeneous cut surfaces with predominantly solid, with variably translucent and hemorrhagic areas; a minor solid nodule corresponding to the nephroblastomatous component was identified (metric ruler is shown for scale). (B) (hematoxylin and eosin, H&E, 4×) In the context of the teratoma, proliferation of an area composed partly of glomerular structures and partly of increased cellularity. (C) (H&E, 10×) poorly differentiated blue round cells interspersed with the outlines of glomerular structures. (D) Immunohistochemistry (IHC) showing positivity for PAX8 supporting their renal immunoprofile. (E) IHC positivity for WT1 consistent with nephroblastic proliferation.

Immunohistochemical characterization of the immature nephroblastomatous component was performed. The blastematous and poorly differentiated tubular elements showed strong nuclear positivity for WT1 and PAX8, supporting renal lineage differentiation. In contrast, these areas were negative for broad-spectrum cytokeratins (AE1/AE3), synaptophysin, and S100 protein, excluding epithelial, neuroendocrine, and neural crest-derived differentiation. These findings were consistent across both surgical specimens and supported the diagnosis of a predominantly mature extragonadal teratoma with a minor nephroblastomatous component (<5% of the overall tumor mass). No extended molecular genetic analyses were performed. Based on histological and molecular characteristics, the final diagnosis was consistent with a predominantly mature extragonadal teratoma containing a minor immature nephroblastic component. A second surgical procedure was performed on the 23rd day after birth to remove the extradural portion of the mass (Figure 3). This entailed a meticulous circumferential dissection of the mass from the sacral plane. The extradural lesion was successfully excised in its entirety, including removal of the coccyx. Histological analysis of the second specimen confirmed the initial diagnosis.

**Figure 3.**
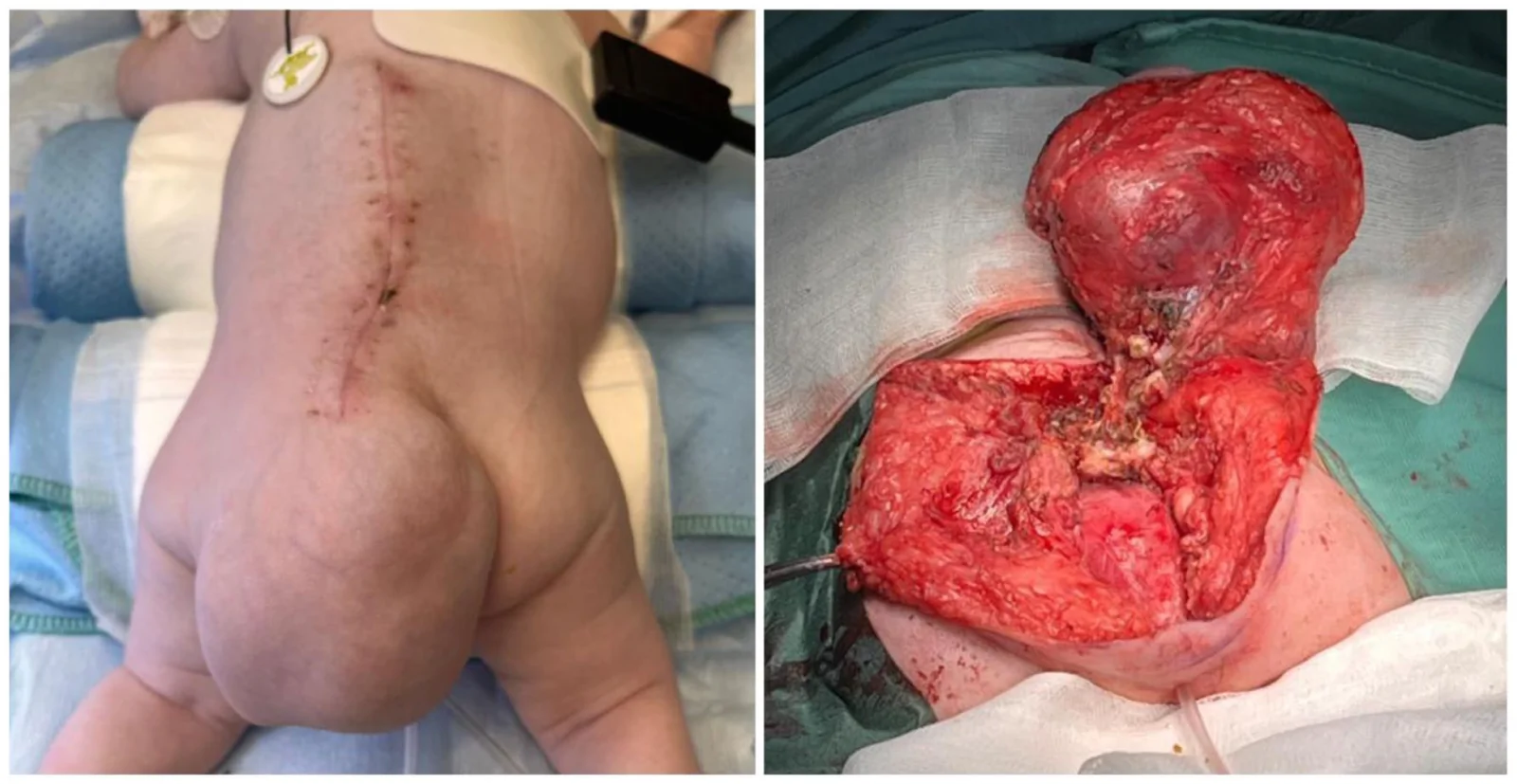
Surgery performed on 29th day of life. Asportation of extradural lesion.

After extraspinal surgery, the neonate displayed a transient paraparesis of the lower right limb, resolving in 3 weeks.

Given the relatively small proportion of nephroblastomatous tissue within an otherwise mature teratoma, a strict radiological and biochemical follow up was initiated. At 8 months of follow-up, neuroradiological assessment showed a stable intraspinal residual disease. Serum AFP levels were within the normal range for age. Clinically, at last follow-up visit the child presented with a right-sided clubfoot, currently undergoing rehabilitation treatment.

## Systematic Review

### Methods

A systematic review was conducted (Figure 4) to identify the clinical presentation, management, and outcome of newborns diagnosed with renal components within sacrococcygeal teratoma. The following search queries: “neonatal teratoma AND nephroblastoma,” “neonatal teratoma AND Wilms,” “sacrococcygeal teratoma AND nephroblastoma,” “sacrococcygeal teratoma AND Wilms,” “sacrococcygeal teratoma AND nephroblastomatosis,” “sacrococcygeal AND teratoma AND EIRT,” “sacrococcygeal teratoma AND ENR,” “Sacrococcygeal AND teratoma AND nephrogenic.” Searches were performed across multiple databases, including PubMed, Scopus, and Web of Science.

**Figure 4.**
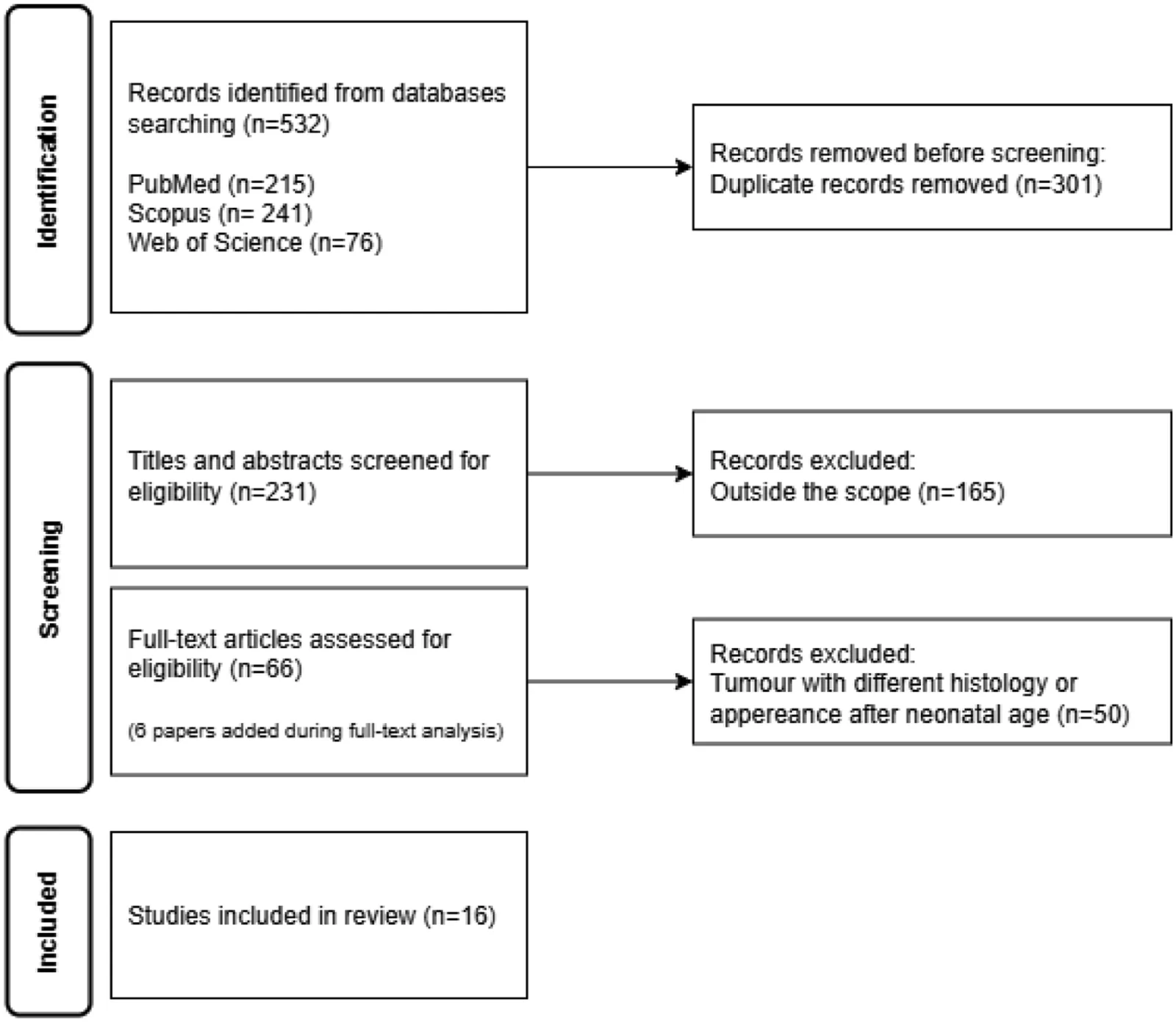
Flowchart for systematic review.

Inclusion criteria were defined as the presence of histological evidence of renal tissue within a teratoma, sacrococcygeal localization and neonatal onset (age: ≤28 days). Exclusion criteria were different histological diagnosis, teratomas located outside the sacrococcygeal region and patients older than 28 days. Primitive intraspinal lumbar teratomas were also excluded from this review.^14,15^ In cases where previous reviews were identified, additional in depth analyses of referenced articles was performed when considered appropriate. Only articles published in English were included.

## Results

A total of 532 of results were identified, of which 301 were duplicates, resulting in 231 different papers. Following abstract screening, independently performed by 2 authors (AC and SR), 165 papers were determined to be outside the scope of the investigation. After full-text analysis of the remaining 66 papers, and with addition of 6 notable papers identified during the review process, 18 eligible patients were identified from 16 articles (with 2 papers describing the same patient). Including the present case report, results are summarized in Table 1.

**Table 1.** Systematic Review of Previous Described Neonatal Patients (<28 d) With Evidence of Renal Tissue in Sacrococcygeal Teratoma.

Reference	Patient n°	Sex	Prenatal evaluation	Age at presentation	Location (dimensions)	Treatment (DOL at S)	Outcome (months after surgery)	Histological diagnosis (as reported)
Sharma et al^7^	1	M	N/A	20 d	SC	S (n/a) + C	NED (8)	Mature teratomatous components and immature component, that is, Wilms’ tumor.
Berry et al^16^	2	F	N/A	14 d	SC	S (n/a)	LR + M (12)	Teratoma. Embryonic, renal tissue adjacent to mucus-producing epithelium
Ward and Dehner^10^ and Tebbi et al^9^	3	F	N/A	Birth^a^ (3 y)	SC, Associated MMC (6 cm × 8 cm × 6 cm)	S (n/a) + R (local, 5400 rads) + C (vincristine + actinomycin-D)	NED (46)	Nephroblastoma arising in a sacrococcygeal teratoma
Andrews et al^17^	4	F	N/A	N/A	SC	S (n/a) + C + R	NED (46)	Immature teratoma with nephroblastic tissue.
Ma et al^18^	5	N/A	24 GW	Prenatal	SC (10 cm × 8 cm × 5 cm)	S (8 d)	NED (8)	Immature solid-cystic teratoma grade I with nephroblastic elements (WT-1+)
Pauniahoet al^8^	6	N/A	N/A	Neonatal	SC	S (<14 d)	NED (48)	Wilms’ tumor within a mature teratoma
Song et al^19^	7	M	Mid-trimester	Prenatal	SC (15 cm × 15 cm)	S (2 d) + C (vincristine and actinomycin-D for 6 mo)	NED (29)	Teratoid Wilms tumor/ teratoma with a predominance of nephroblastic elements
Mickle and McLennan^20^	8	F	N/A	Birth^a^ (11 y)	SC Associated MC + M at 11 y.o.	S + CT (actinomycin-D, vincristine Cyclophosphamide, and adriamycin)	(N/A)	Malignant teratoma arising within a lipomeningocele, final pathological diagnosis was Wilms’ tumor.
Gupta et al^21^	9	F	N/A	Birth	SC + Intrapelvic (9 cm × 7 cm × 4.5 cm)	S (9 mo)	N/A	Immature teratoma with [. . .] renal differentiation
Al-Adnani et al^22^	10	F	N/A	Birth	SC	S (15 mo)	NED (9)	Sacrococcygeal teratoma with extensive nephrogenic differentiation (WT-1 +)
Ma et al^23^	11	M	N/A	10 d	SC (6 cm)	S (N/A)	NED (154)	Teratoma with ENR (<1%)
	12	M	N/A	8 d	SC (8 cm)	S (N/A)	NED (46)	Teratoma with ENR (<1%)
	13	F	N/A	15 d	SC (8 cm)	S (N/A)	NED (10)	Teratoma with ENR (5%)
Heerema and McKenny^24^ (2005)	14	N/A	N/A	Birth	SC (3.8 cm)	S (40th GW)	NED (25)	Immature teratoma with focal presence of immature nephrogenic tissue
Bhimreddy et al^25^	15	F	N/A	8 d	SC Associated with tethered spinal cord	S (6 mo)	N/A	In the context of LMM: NR tissue or, less likely, a teratoma with renal differentiation.
Valdiserri et al.^26^	16	F	N/A	Birth	SC (9 cm)	S (3 d)	NED (72)	Immature Sacrococcygeal Teratoma (G3) with renal predominant immature component
	17	F	N/A	N/A	SC (2 cm)	S (18 mo)	E^b^ (36)	
Patra et al^27^	18	M	N/A	Birth	SC + intrapelvic (12 cm)	S (N/A)	NED ^c^ (30)	Immature teratoma (G2) [. . .] with immature renal tissue composed of glomeruli, tubules, and blastema.
Our case	19	F	29 GW	Prenatal	SC + Intrapelvic	S (5.23)	RD (4)	Predominantly mature extragonadal teratoma with minor nephroblastomatous component (<5%).

Abbreviations: S, surgery; C, chemotherapy; R, radiotherapy; NED, “No Evidence of Disease”; RD, residual disease; LR, local recurrence; M, metastatic disease (lung); E, Exitus; GW, gestational week; SC, Sacrococcygeal; MC, meningocele; MMC, myelomeningocele; LMM, Lipomyelomeningocele.

a The lesion was identified at birth, and a histological diagnosis was subsequently obtained at surgery (indicated age).

b Exitus secondary to acute broncho-pneumonia and an obstructed A-V shunt.

c Patient underwent second surgery for left testis tunical gliomatosis (6 mo after first surgery).

Among the 19 patients (including the present case), 11 were females, 5 were males, and sex was not reported in 3 cases. Prenatal evaluation was performed in only 3 patients (15.7%). Neural tube defects were associated with 3 patients (including tethered spinal cord, lipo-myelomeningocele, and meningocele).

All patients underwent surgical resection (n = 19) with subsequent chemotherapy administered in 5 cases (26.3%). When reported, a regimen of vincristine and dactinomycin was preferred. One patient (Case 8) additionally received cyclophosphamide and adriamycin. Two patients underwent radiotherapy (10.5%).

Regarding histological findings, in the context of SCTs, renal components were diagnosed as nephroblastoma/WT in 4 cases (21%) and as ectopic nephrogenic rests (ENR) or extensive nephrogenic differentiation in 15 cases (79%).

The median follow-up time after surgery was 29.5 months (range: 4-154 months). Local recurrence with evidence of lung metastases occurred in 1 patient (Case 2, 5.2%) 24 months after surgery; this patient had no subsequent follow-up data available. One patient (our case, 5.2%) is undergoing longitudinal monitoring for residual disease. Additionally, metastatic disease was identified in 1 patient at the time of delayed surgery (case 8).

Death was reported in only 1 patient (Cases 17, 5.2%), attributed to bronchopneumonia and obstructed arteriovenous shunt complications. Follow-up data were unavailable for 2 patients (10%). Excluding the present case report and previous detailed patients, all other individuals were classified as having no evidence of disease (NED) at last follow-up.

## Discussion

The presence of somatic renal differentiation within SCTs represents a very rare occurrence, posing unique diagnostic and therapeutic challenges. Given the absence of clear histological criteria to define these components and predict clinical behaviour, various terms have been used in the literature, including ectopic immature renal tissue (EIRT), ectopic nephrogenic rests (ENR), extrarenal nephroblastomatosis, or directly nephroblastoma/WT. Histological criteria to differentiate EIRT from nephroblastoma-like components have been proposed.^12^ Specifically, cytological features (such as marked atypias, atypical mitoses, or pleomorphism) and structural features (expanding, spherical nodules with pseudocapsule formation) should be considered consistent with nephroblastoma. Conversely, small multiple nests or islets of renal cells, even in the presence of mild atypia of high mitotic rates, should be regarded as immature renal tissue.^12^

In the context of testicular germ cell tumors, Micheal et al^28^ proposed additional criteria (specifically, nephrogenic components occupying half to an entire microscopic field seen with a 4× objective) to define nephroblastoma rather than immature renal components. However, no studies have specifically examined ultrastructural characteristics or protein expression profile to differentiate these components.

EIRT is considered to have degenerative potential toward extra-renal WT,^11,12^ while other Authors state that ERWT may arise from within teratomas.^13^ Furthermore, the definition of “Teratoid Wilms tumor” (TWT) has been used to describe a nephroblastoma variant featuring more than 50% heterologous tissues within the tumor mass.^29^ TWT was strongly associated with younger age at presentation and bilateral renal involvement.^30^ A further proposed distinction from stromal WT is the presence of ≥3 heterologous elements (HEs).^30^ When located outside the kidney, Song et al^19^ suggested that TWT may originate from a teratomatous lesion.

SCTs contain elements of all 3 germ cell layers^1^ and several theories have been proposed regarding their pathogenesis, including error in the germ cells migration, dysregulation of the transcription factor involved in germline specification, or failure of residual cells to undergo apoptosis.^3^

The complex interplay between the histological components of EIRT, WT, and SCTs, along their relation to cell differentiation processes, renders differential diagnosis highly challenging. In the absence of established guidelines for the management of this rare condition, we conducted a systematic review to describe clinical presentation and therapeutic approaches.

Prenatal assessment is critical and should guide careful pregnancy management and birth planning. Currently, diagnosis is often made during routine ultrasound scans between 18 and 20 weeks of gestation.^4^ Due to high vascularization of SCTs, the fetus is at risk of developing systemic shunts, potentially leading to high-output heart failure (HOHF).^5^ In cases of suspected SCT, specialized echocardiographic evaluation should be performed to detect for early signs of high-output state.^6^ Tumor volume-to-fetal weight ratio has also been proposed as a prognostic tool.^6^ However, the prognosis remains poor in the presence of HOHF and fetal hydrops,^5^ especially if onset occurs before the 28th week of pregnancy.^6^ Serial ultrasound monitoring is considered mandatory in fetuses diagnosed with SCTs. In the absence of signs of abnormal fetal growth and development, delivery should occur near term in a tertiary referral center.^5^ Cesarean section is recommended for tumors larger than 5 cm in diameter, as vaginal delivery increased the risk of hemorrhage secondary to tumor rupture. Blood transfusion resources should be readily available in the delivery room.^5^ In very high-risk patients, ex utero intrapartum treatment (EXIT) procedures may be considered.^1^ Despite its recognized importance, prenatal assessment is reported in only a minority of cases in the literature, likely due to the unavailability and lack of standardization of antenatal ultrasound monitoring

Postnatal management of SCTs is based on complete tumor excision, including removal of the coccyx.^2,3^ Complete resection should be considered imperative, as incomplete surgery, particularly in cases with intrapelvic extension,^5^ is associated with higher risk of recurrences, including development of yolk sac tumor.^31^ Preoperative angiography with embolization has been proposed to minimize surgical risks.^32^

From a molecular-pathological perspective, the characterization of nephrogenic components within sacrococcygeal teratomas remains challenging. In the present case, immunohistochemistry provided the main biological insight, with WT1 and PAX8 expression confirming nephroblastic differentiation. Extended molecular analyses typically employed in intrarenal Wilms tumor—such as assessment of WT1 or CTNNB1 mutations, 11p15 imprinting abnormalities, or other recurrent genetic alterations—were not performed. This represents a limitation of the study and was mainly related to the extremely limited extent of nephroblastomatous tissue, its complete integration within a predominantly mature teratoma, and the neonatal clinical setting.

Nevertheless, current evidence suggests that, in extrarenal and teratomatous settings, molecular alterations characteristic of Wilms tumor are inconsistently reported and their clinical significance remains uncertain. In this context, an integrated diagnostic approach combining histoarchitectural evaluation and immunophenotypic profiling remains central. In our case, the absence of overt cytological atypia, the small volume of nephroblastomatous tissue, and the supportive immunoprofile favored a conservative diagnostic interpretation and informed a management strategy based on complete surgical excision and close longitudinal surveillance rather than immediate adjuvant therapy.

Serum alpha-fetoprotein (AFP) levels were elevated in our patient; however, AFP concentrations are physiologically high in neonates and may overlap with values observed in malignant conditions. As highlighted by Głowska-Ciemny et al,^33^ this physiological elevation represents a major diagnostic pitfall in early infancy. In our case, no definitive yolk sac tumor component was identified. The nephroblastomatous focus was extremely limited and identified in a single histological section; this focus was exhausted on deeper levels, precluding further immunohistochemical studies, including AFP staining. Importantly, no morphological features suggestive of yolk sac tumor were observed, and no additional malignant germ cell elements were detected despite extensive sampling, underscoring the limitations of AFP interpretation in neonatal sacrococcygeal teratomas.

Following surgery, close clinical and radiological follow-up, along with periodic monitoring of serum AFP levels, should be instituted.^33^ However, it is crucial to remember that AFP levels are physiologically elevated in the neonatal period, with values up to 100 000 ng/ml considered normal.^34^

In our review, all patients underwent surgery at highly variable times, ranging from the first week of life to later childhood (3 and 11 years of age). These latter cases were included due to the presence of lumbosacral masses identifiable at birth. In our patient (case 19), complete surgical excision was unfeasible due to important intraspinal component; therefore, strict radiological and biochemical monitoring was initiated. Regarding chemotherapeutic, no universally accepted clinical indication currently exists. Previous medical regimens, mainly involving D-actinomycin and vincristine, have been administered in patients with reported histological diagnosis of nephroblastoma arising within SCTs or TWT located in the sacrococcygeal region. Notably, specific criteria defining malignancy have not been reported. Radiotherapy was administered in 2 patients.

Given the excellent prognosis in the reviewed cohort, we propose that adjuvant medical therapy should be reserved for highly selected patients with consistent risk factors (e.g., histological evidence of malignancy or presence of metastatic disease). A conservative “wait and see” strategy should be adopted for patients with renal components identified within completely excised SCTs.

As demonstrated in our case, significant neurological comorbidities can be present at diagnosis or following SCTs management underscoring the importance of early initiation of physical therapy and prompt neurological referral.

This review is limited by the retrospective nature of data collection, the heterogeneity of histological descriptions, and the lack of direct access to pathological specimens for second review. Furthermore, some data were extracted from articles not primarily focused of renal tissue occurrence (e.g., national tumor registers or case series), potentially introducing reporting bias.

In cases reported in the literature as Wilms tumor arising within sacrococcygeal teratomas or other extrarenal sites, molecular genetic data are rarely available. Most diagnoses have relied predominantly on histomorphological criteria, including expansile nephroblastomatous growth, cytological features of malignancy, and in some cases the presence of a fibrous pseudocapsule, rather than on molecular profiling.^9,10,35^ This reflects both the historical nature of many reports and the technical limitations related to small tumor volume and rare presentation. Consequently, it remains uncertain whether extrarenal Wilms tumors arising in teratomatous contexts share the same molecular alterations described in intrarenal Wilms tumor.^36^ Recent reports of extrarenal and teratoid Wilms tumors continue to emphasize the central role of morphology and immunohistochemistry, while highlighting the need for systematic molecular studies to clarify pathogenetic relationships and potential prognostic implications.^35,36^ This represents a promising avenue for future multicenter investigations in these exceptionally rare tumors. Nevertheless, this review presents a cohort of neonatal SCTs with evidence of renal tissue described to date and may represent an important reference for future research in this rare clinical entity.

## Conclusions

The occurrence of renal tissue within SCTs represents an extremely rare condition, leading to complex diagnostic and therapeutic challenges. The spectrum of renal components in these specimens may vary widely, and no clear management guidelines have been published to date. Differentiation between ectopic immature renal tissue (EIRT) and true malignant components, such as nephroblastoma-like lesions, remains challenging, even in the presence of proposed histopathological criteria. Surgical resection continues to represent the hallmark of treatment; however limited data are available regarding the role of additional therapeutic interventions, such as chemotherapy. Given the potential association with other anatomical abnormalities and the risk of neurological comorbidities, a comprehensive and multidisciplinary evaluation should be pursued in newborns diagnosed with renal components in the setting of SCTs.
